# The role of taxation measures in the management of harmful products, services, and practices in Iran: a qualitative study

**DOI:** 10.1186/s12889-022-14673-w

**Published:** 2022-12-09

**Authors:** Mahdi Kooshkebaghi, Hossein Dargahi, Sara Emamgholipour

**Affiliations:** 1 Department of Health Management and Economics, School of Public Health, Tehran University of Medical Sciences, Tehran, Iran; 2 Department of Health Management and Economics, School of Public Health, Health Information Management Research Center, Tehran University of Medical Sciences, Tehran, Iran

**Keywords:** Tax, Harmful products, Management, Improving health, Financing, Qualitative study

## Abstract

**Background and aim:**

Levying a tax on harmful products, services, and practices can affect consumer choices, effectively preventing diseases and reducing health care costs. The goal of this study was to investigate the role of taxation as a powerful financial tool in the management of harmful products, services, and practices to maintain and improve public health and preserve the financial sustainability of the health care system.

**Materials and methods:**

This qualitative study was conducted in 2020–2021. In order to collect information for this study, semi-structured interviews were conducted. Using purposive and snowball sampling methods, 38 managers, policymakers, economists, and key experts were interviewed. Data were analyzed using the content analysis method. The transcribed interviews were further imported into MAXQDA for classification, and relevant codes were extracted.

**Findings:**

In this study, 6 main themes and 19 subthemes were labeled. The main themes included 1) objectives, effects, and requirements of the taxation of harmful products, services, and practices, 2) definition, instances, elasticity, and grading of harmful products, services, and practices, 3) Problems in controlling harmful products, services, and practices, 4) controlling harmful products, services, and practices, 5) traffic violations and accidents, and social harms, and 6) tax revenue use and the share of health care. The effects of taxing harmful products include reduced access to these products, reduced demand for harmful products, and the promotion of public health.

**Conclusion:**

Harmful products, services, and practices have major health and financial implications for individuals, families, and society. To improve public health, the demand for these products and services can be controlled through taxation measures to push consumers toward less harmful alternatives.

## Introduction

In recent years, with the spread of non-communicable diseases (NCDs), health has been threatened and great damage has been done to the health and economy of countries [1, 2]. NCDs are resulting in more than 41 million deaths per year, which is 71% of global mortality [3–5]. These diseases are mainly caused by harmful foods and drinks, smoking, and alcohol consumption [6]. Harmful products and services have reduced the health level of people and imposed the cost of being unhealthy and disease on individuals, families, and society. The consumption of these products and services has increased significantly in the last three decades [7–9]..

Fiscal instruments, such as taxes or subsidies, can promote healthy behaviors [10–14]. The most important impacts of a higher tax on unhealthy products will be a fall in the demand for these products, and the promotion of healthy lifestyles, which in turn reduces the negative health implications of these products and the ensuing care and treatment costs [15–18].

Imposing taxes on harmful products, services, and practices, does not only improve public health [19, 20] but also provides revenues to governments [21–24]. These revenues can be used for the sustainable financing of health systems [25] as well as other sectors. The results of a study showed that tax revenue had a positive significant impact on Iran’s health costs between 1985 and 2015 [26]. According to the results of another study, income had a positive effect on healthcare expenditures during 1995–2014 [27].

A study by the World Health Organization (WHO) has shown that a 10% growth in the price of harmful goods reduces the consumption of these goods, which is 4% in developed countries and 8% in developing countries [28]. Many countries have implemented plans to introduce taxes on Sugar-Sweetened Beverages (SSBs) and foods high in salt, fat, and sugar [29–37]. Also, tobacco taxation policies have been implemented to improve health in many countries [38–44]. In this regard, tobacco taxes have caused a 12% reduction in sales in Turkey between 2008 and 2012, and a 50% reduction in sales in France between 1990 and 2005 [45].

A study in South Africa similarly demonstrated that taxation on sugary drinks could prevent 72,000 deaths and the loss of 550,000 years of healthy living, as well as reduce health expenses by $5 billion [46]. Also, according to a study in Vietnam (2018), increasing taxes on tobacco prevented the loss of $57 billion of resources [47].

In Iran, despite the high consumption of harmful goods, such as tobacco, the prices of these products and taxes on them have not increased much. For example, the total share of tobacco tax as a percentage of the retail price is only 21.7% [48] which is far below the minimum of 75% recommended by the WHO [49]. In addition, evidence in Iran has shown that the incidence of obesity, overweight, hypertension and cardiovascular diseases has increased significantly, of which the consumption of unhealthy foods and beverages has been one of the most important factors [50]. Notwithstanding, very few studies [7, 51, 52] have been done on Iran about the role of taxes in controlling the consumption of harmful products and services with less attention paid to harmful practices. Traffic violations and accidents, and environmental pollution by individuals and factories, are among the most important harmful practices that threaten health.

Moreover, most studies on taxes on harmful goods have been done quantitatively, and qualitative studies in this field are very limited. Meanwhile, qualitative research enables a deeper understanding of experiences, phenomena and context, and helps to extend knowledge and understanding of realities [53]. Accordingly, using a qualitative approach, this study aimed to investigate the role of taxation as a powerful economic tool in the management of harmful products, services, and practices in pursuit of the primary goals and functions of the health care system (i.e., maintaining and improving public health and preserving the financial sustainability of the health system) in Iran.

## Materials and methods

### Study design

This study was conducted in 2020–2021. The study was performed with a qualitative approach to gain comprehensive, deep, and rich information on the subject. In this study, we followed the Consolidated Criteria for Reporting Qualitative Research (COREQ) Checklist [54].

### Participants and setting

The statistical population of this study consisted of managers, policymakers, deputies, and key experts from the Iran National Tax Administration (INTA), the Ministry of Health and Medical Education, the Ministry of Economic Affairs and Finance, the Plan and Budget Organization, Department of Environment, members of the parliament (MPs), as well as professors and key experts from several reputable universities in Iran. The inclusion criteria in this study included at least a master’s degree (Fields such as health management, health policy, health economics, as well as non-health economics and management, food sciences and industries, environment and public health) or a medical doctorate (MD), and showing a willingness to participate in the interview. The sample size of the study was arrived at based on data saturation and to obtain the most information about the subject. In total, 38 managers, deputies, policymakers, and key experts were interviewed upon obtaining their consent. Both purposeful and snowball sampling methods with maximum variation were also employed. After coordination, most of the interviews were conducted at the respondents’ workplace, and some were completed by phone calls as wished by them (32 and 6 cases were conducted in person and by phone calls, respectively).

### Data collection

The initial interview questions were identified using tax rules/guidelines in budget laws and country development programs, research background (studies conducted in Iran and other countries), and expert opinions. To ensure the validity and significance of the questions, pilot interviews were conducted with three members of the statistical population who were not among the selected participants. The type and order of the questions were determined, and unintelligible questions were revised; ultimately, the final interview questions were developed. The interviews were conducted in Persian and in a semi-structured manner with open-ended questions and by prior appointment over nine months. The interview sessions were also recorded with the permission of the interviewees. The interviews were transcribed after the end of each session in order to become aware of the data saturation time and increase their accuracy.

### Trustworthiness of data

The robustness, accuracy, and precision of the data were further determined based on the four criteria of credibility, transferability, dependability, and confirmability [55]. To ensure the credibility of the data, member checks and peer debriefing (these members were part of the sampled respondents, but peers were not part of it), allocation of enough time for data collection, and researchers’ long-term involvement (continuous and regular review of data collected from interviews) were used. The transferability of the data was also provided through interviews with different informants from various departments (these informants were part of the sampled respondents) and a detailed description of the data. To ensure the dependability criterion, the study process was submitted to quality consultants, and they approved the study results after some reviews. These reviews included data collection, sampling, preparation of interview guide and questions, how to conduct interviews, data saturation, transcribing interviews, and data analysis. Given that the whole process of the study was carried out with the guidance of two qualitative studies consultants, no change was made to the original data collected. To meet confirmability, indicating the stability of the data (along with observing the researchers’ impartiality), peer cheeks (these peers were not part of the sampled respondents), and a review of manuscripts was used. The interviews, coding, and the extraction of the main themes and subthemes were fulfilled by two members of the research team who did not have any conflict of interest with the research subject.

### Data analysis

To analyze the data, the content analysis method was used. After transcribing, categorizing and coding the interviews, the final text was translated into English by a reputable translation agency. Also, the translated text was checked by the researchers of this study. Then, the transcriptions were read several times to gain a general understanding. In addition, the transcribed interviews were sent to some interviewees to add or subtract some issues as needed. Afterwards, the transcribed interviews were entered into MAXQDA version 12 for classification, and the initial codes were extracted. Then, the final codes and themes were obtained with the cooperation and comments of the members of the research team (The authors of this study) and in consultation with interviewees (including several university professors and tax managers); finally, the codes were grouped into main themes and subthemes.

### Ethical consideration

Before the interviews, the research objectives (along with the arrangements required to maintain the confidentiality of the data) were presented orally to all interviewees, and written consent was obtained. Considering that the coordination of all the interviews was done in person, in the telephone interviews, the consent form was provided to the interviewee a few days before the interview, and after completing the form, the researcher received the form in person. Study participants were reassured that the interview data would only be used for this study and would not be shared with others. The participation of the interviewees in the research was completely voluntary, and these people could opt out of the interview at any time. Also, no problem was observed in evaluating the risks and benefits of this research. Assessment of risks and potential benefits is necessary to determine that a research study is ethically acceptable and would protect participants [56]. In face-to-face interviews, to prevent the transmission of the COVID-19 infection, while maintaining a distance of at least 2 m, interviewers and respondents used masks.

## Results

According to the transcribed interviews and a review of relevant texts, six main themes and 19 subthemes were identified in this study (The main themes included, 1) objectives, effects, and requirements of the taxation of harmful products, services, and practices, 2) definition, instances, elasticity, and grading of harmful products, services, and practices, 3) problems in controlling harmful products, services, and practices, 4) controlling harmful products, services, and practices, 5) traffic violations and accidents, and social harms, and 6) tax revenue use and the share of health care. Most of the interviewees were from the INTA, Tehran University of Medical Sciences, and the Ministry of Health and Medical Education. The information about the place of employment, work experience of the interviewees, and demographic information are illustrated in Tables 1 and 2. Since the statistical population of this study, especially policymakers, managers, and deputies of relevant organizations, were mostly men, most of the interviewees (36 people) were men, and only two interviewees were women

**Table 1:** Place of employment and work experience of the interviewees

Interviewee No	place of the employment	Work experience (years)
1	Tehran University of Medical Sciences	20
2	Tehran University of Medical Sciences	40
3	Tehran University of Medical Sciences	20
4	Tehran University of Medical Sciences	6
5	Tehran University of Medical Sciences	6
6	Tehran University of Medical Sciences	46
7	Tehran University of Medical Sciences	16
8	Iran University of Medical Sciences	35
9	Shahid Beheshti University of Medical Sciences	12
10	University of Tehran	21
11	University of Tehran	12
12	Allameh Tabataba’i University	20
13	Plan and Budget Organization	22
14	Plan and Budget Organization	16
15	Ministry of Health and Medical Education	21
16	Ministry of Health and Medical Education	26
17	Ministry of Health and Medical Education	13
18	Ministry of Health and Medical Education	6
19	Ministry of Health and Medical Education	12
20	Ministry of Health and Medical Education	23
21	Ministry of Health and Medical Education	22
22	Islamic Parliament of Iran	31
23	Islamic Parliament of Iran	35
24	Islamic Parliament of Iran	15
25	Ministry of Economic Affairs and Finance	20
26	Department of Environment	27
27	Iran National Tax Administration	31
28	Iran National Tax Administration	24
29	Iran National Tax Administration	13
30	Iran National Tax Administration	21
31	Iran National Tax Administration	27
32	Iran National Tax Administration	33
33	Iran National Tax Administration	26
34	Iran National Tax Administration	32
35	Iran National Tax Administration	25
36	Iran National Tax Administration	38
37	Iran National Tax Administration	25
38	Iran National Tax Administration	16

**Table 2:** Demographic information of the participants

Variable	Sub-variable	Count	Percentage
Gender	Male	36	94.74
	Female	2	5.26
	Total	38	100
Age (Years)	30–40	9	23.68
	41–50	10	26.32
	51–60	15	39.47
	Above 60^a^	4	10.53
	Total	38	100
Work experience (Years)	6–10	3	7.90
	11–20	13	34.21
	21–30	13	34.21
	Above 30	9	23.68
	Total	38	100
Level of education	Masters	13	34.21
	Medical	5	13.16
	PhD	20	52.63
	Total	38	100

^a^In general, the retirement age in Iran is 60 years for men and 55 years for women. Retirement conditions for university professors include at least 65 years of age or 30 years of work experience

### Objectives, effects, and requirements of the taxation of harmful products, services, and practices

#### Objectives of taxation

Before levying taxes on harmful products, services, and practices, first, the exact goals of this taxation must be determined. People interviewed in this study believed that the two main goals of this taxation should be to promote public health and ensure the financial sustainability of the health care system. Other mentioned goals included improving production (using healthier raw materials, modernizing equipment, and modifying the production process), correcting behavioral habits (avoiding smoking, and avoiding dangerous driving and violent behaviors), and reducing consumption of unhealthy foods and drinks. Modernizing equipment means buying and using new and advanced equipment, and modifying the production process means how to combine healthier raw materials. In this regard, interviewee 20 believed that:

“Taxing harmful goods has two purposes: One is that rising taxes and prices of such products will reduce their consumption rates, and this will promote public health; next, the tax received is a sustainable source of funding for the health system.”

#### Effects of taxation

The positive effects of taxing harmful products include reduced access to these products, reduced demand for these products, revenue generation, change in consumption patterns, change in production lines, and increased production of less harmful alternatives. However, these taxes may also have some negative consequences. Interviewee 17 stated that:

“Taxing such products has two benefits. Firstly, it makes them more expensive, which means some people will not be able to afford them or not as much. Secondly, it brings revenue, which can be earmarked to the health care system.”

Further, according to interviewee 7:

“With a tax hike, for example, on cigarettes, some families, especially those that are poorer, will have to cut their main expenses to afford cigarettes.”

#### Requirements of taxation

According to most interviewees, the most important requirements for this taxation are determining the exact tax rates by paying attention to anticipated effects and different aspects of the issue, culture building, raising public awareness, and paying attention to the effects of price changes on different income groups. In this regard, interviewee 6 said:

“We should be very careful in putting taxes on harmful products and consider everything before changing the rates. The important thing is to carefully choose the exact rate and how long and for which groups it will be applied.”

### Definition, instances, elasticity, and grading of harmful products, services, and practices

#### Definition and instances

To better control harmful products, services, and practices through taxation for health promotion and financial sustainability purposes, these products, services, and practices need to be properly defined with instances. Interviewee 18 believed that:

“Our definition of harmful products must be expanded. We need to increase the tax base for such goods, not just increase the tax rates. There are so many products that have health impacts but are not taxed.”

#### Elasticity

One of the most important issues that need to be considered when discussing taxation is the elasticity of demand for products and services. For products with low price elasticity, demands tend not to change significantly with price changes, especially in the long run. Thus, for these products, price tools need to be utilized alongside other strategies. Regarding elasticity, some interviewees stated:

“When talking about taxing harmful products, you should consider which groups of people use these products the most, the poor or the rich. Then, you should determine the elasticity or the reaction to prices.” (Interviewee 5)

“Once we know the elasticity of different products, we can very well predict the effectiveness of taxes in reducing their consumption and changing people’s consumption habits and then formulate more effective tax and pricing policies.” (Interviewee 25)

#### Identification and grading of harmful products, services, and practices

Since different harmful products and practices will have different levels of health implications, they must be graded based on the type and severity of their effects. The amount of tax levied on each type of product or service should be proportional to the severity of its effects. In this regard, interviewee 13 believed that:

“Harmful products must be classified. For products that are extremely harmful, production and distribution should be outrightly banned. For goods and services that are harmful but are too commonly used among the people and the severe consequences of prohibition (including, the increase in the consumption of other harmful goods due to the lack of suitable alternatives, the increase in smuggling of goods due to poor supervision, and the production of fake and low-quality goods), we must take less coercive actions; for example, use the taxation to gradually eliminate the market of these goods. For those products that have proven adverse health effects, but their effects are more long-term than immediate, taxes should be collected to reduce consumption.”

### Problems in controlling harmful products, services, and practices

#### Poor decision making, planning, and execution

According to the interviewees, the most important challenges in decision making, planning, and implementing a tax policy for controlling harmful products, services, and practices include the complexity of the tax system, the non-deterrence of taxes, the relatively poor coordination and cooperation of the country’s legislative and executive bodies, the multitude of government bodies in charge of dealing with the issue, and the excessive focus on certain harmful products (such as tobacco and carbonated beverages) and neglect of other harmful products.

Inexact and outdated tax codes, poor taxing structures, the lack of enough information about harmful products and services, poor commitment to deal with the issue, inadequate monitoring and supervision, conflicts of interest, and the lack of specific job descriptions in government bodies for controlling harmful products, services, and practices, were other challenges and problems in controlling harmful products, services, and practices. In this regard, interviewee 8 added that:

“The excessive consumption of harmful goods and the increase in related diseases and their injuries are in part due to incorrect tax policies and pricing. Weaknesses in the enforcement of regulatory and control laws and poor tax structures are two important reasons in this respect.”

#### Production-related problems

The interviewees stated that imposing a tax on products will increase their prices and cause problems for manufacturers. For example:

“When we increase taxes, the increased cost of the product will reduce the demand, which will damage the manufacturer. Naturally, manufacturers have to lower their prices to prevent sales drop.” (Interviewee 15)

#### Smuggling and poor supervision

Although the impact of a tax increase on the smuggling of taxed products into the country needs to be considered, there is not much evidence about this impact. The interviewees believed that poor supervision would be a serious problem in managing the effect of taxes on harmful products. Interviewee 24 believed that:

“Experience and scientific evidence show that smuggling is not usually caused by tax policies or the rising prices of domestically produced products. The source of the illegal trade of harmful products can be traced back to legal, administrative, and regulatory inadequacies, government’s poor capability in managing taxes, poor economic conditions, poor quality of some domestically produced products, and even cultural problems.”

#### Increased consumption of harmful products

Taxing some harmful products or raising their prices may lead to the increased consumption of other harmful products or the increased incidence of certain risky behaviors due to reduced access to the original products. Regarding this problem, an interviewee said:

“After taxing harmful products, the market will be full of low-quality alternatives and counterfeits, which could be even cheaper and more damaging than the originals.” (Interviewee 2)

#### Conflict of interest

Since some individuals, organizations, and businesses greatly benefit from the production, distribution, and trade of harmful products, conflicts of interest will be a major obstacle to the implementation of higher taxes on many of these products. In the context of profit-seeking motive of some people and groups, it was stated that:

“Rising taxes on harmful products may hurt the economic interests of some people. So, they will do everything they can to prevent it.” (Interviewee 27)

### Controlling harmful products, services, and practices

#### Imposing taxes, duties, and price hikes

According to most participants interviewed in this study, one of the best ways to control harmful products and practices is to levy higher taxes and duties and raise their prices. Of course, the tax increase must be purposeful and proceed in stages and can be reduced or reversed upon reaching the envisioned objectives about products or production processes. Interviewee 1 stated that:

“To reduce the consumption of harmful goods, I have to make them harder to achieve, I will make them so expensive that, firstly, people cannot buy harmful goods, and secondly, if they consume them and the disease is created, I will compensate the treatment costs from the consumption tax.”

Further, interviewee 30 added that:

“There are so many harmful products, and their share in the consumer goods should be reduced. We have to tax them. These taxes should increase to the point that people can no longer afford them.”

#### Providing alternatives

According to some interviewees, imposing higher taxes on harmful products will boost the production of healthier or less harmful products. Tax breaks and discounts can also be used to encourage the production and use of high-quality, healthier alternatives. For example, it was said:

“If we work more on alternatives, imposing taxes and price hikes on harmful products can boost industries that make healthy products, which will also boost jobs in that sector.” (Interviewee 24)

#### Attention to all determinants of supply and demand

Demand and supply of harmful products can be a function of many factors. Thus, before adopting tax and non-tax measures for controlling them, the reasons why these products are consumed should be determined. In addition to being a function of price, demand for harmful products and services is also a function of beliefs, habits, advertising, and product quality. Interviewee 8 said:

“For our tax measures to be effective, we need to influence consumer choices and decisions. Unfortunately, not much is being done on this issue. If we work on the pricing and quality of our products or their appearance, then demand and consumption will improve.”

### Traffic violations and accidents, and social harms

#### Fining and taxation

Traffic accidents are a major cause of injury, disability, death, and rising health care costs in Iran. Since drivers, automakers, and government bodies (such as municipality, police, and highway administration) all play a role in the incidence and severity of traffic accidents, the role of each of these actors in the accident or damage should be correctly determined and according to that, taxes, fees, or fines should be received from the offending persons or organizations. Social harms and behavioral offenses that adversely affect the health of others can also be fined. However, to do so, first, detailed studies should be conducted on the subject to fully explore its legal and executive implications. Interviewee 6 stated that:

“In my opinion, it is not only appropriate but also necessary to put a tax on driving violations and social deviances, those behaviors that have external consequences. If someone has caused an accident or violated the law, is guilty, and his actions have been preventable, he must pay a fine because his behavior has harmed society and others.”

#### Deterrence of fines and taxes

Before imposing fines, fees, or taxes on traffic violations and social deviancies that threaten the health of others, the deterring effects of these measures should be carefully considered. For this purpose, it is necessary to consider different aspects and conditions of the violations and examine the effects of fines and taxes at different time intervals. Interviewee 31 believed that:

“You can put taxes on traffic violations and social deviances, but it is important to determine how much deterrence they create; whether you have increased the cost of the violation. This fine should prevent a person from repeating the violation or at least reduce the probability.”

#### Requirements and conditions for fining and taxation

The conditions and requirements for levying fines and fees on traffic violations and accidents and social deviances include access to enough data about the violations and their root causes, a clear and specific vision of the purpose of fines, an exact assessment of legislative measures for implementing fines and taxes, expert meetings with the participation of relevant individuals and organizations, a good assessment of the effects and consequences of the implementation of such fines and taxes, using the experiences of other countries in this area, and considering the social, cultural, and economic conditions of the country. In this regard, interviewee 36 believed that:

“Firstly, it should be clear what the goal of taxing these risky measures is. Secondly, we should also pay attention to the effects of taxation and fining. Thirdly, how can a law be made for these violations and how can it be implemented.”

Also, interviewee 15 stated that:

“In such cases, we need to discuss two issues. One is that we want to reduce deviances. The other is how such a tax can even be collected. We need systems with enough sensitivity and intelligence to detect them.”

### Tax revenue use and the share of health care

#### Use of revenues from the taxation of harmful products, services, and practices

According to most interviewees, the bulk of revenue from the taxation of harmful products and practices should be allocated to the health care sector. Of course, this does not necessarily mean that the revenue should be given to the Ministry of Health, as other government agencies that contribute to public health can also use the revenues for health promotion purposes. The most important areas where these taxes revenues can be utilized include promoting preventive health, improving access to services, improving treatment coverage, modernizing and improving production processes, and boosting high-quality, healthy alternatives. Interviewee 23 added that:

“Taxes on harmful goods can be used to help industries produce better and healthier goods or acquire healthier raw materials at better prices. These taxes can also be used to treat related diseases. For example, you can use the duties and taxes on harmful products to create a fund.”

#### Use of revenues from the taxation of advertising harmful products and services

Some of the interviewees mentioned the possibility of levying taxes on the advertisement of harmful products and services. The revenue gained from this tax can be used for culture building, improving people’s eating habits, and other such initiatives. One may also impose higher taxes on certain products and also certain advertising processes that are deemed more harmful. For example, it was stated that:

“It is important to receive taxes and duties from advertising of harmful goods. Revenues from this type of taxes can be invested to improve the quality of the products, health measures, and disease prevention.” (Interviewee 9)

## Discussion

Harmful products, services, and practices tend to have major health and financial implications for individuals, families, and society. The demand for these products and services can be reduced by using taxation measures to raise their prices and encourage consumers to use less harmful alternatives: an approach that can contribute to public health and help maintain the financial sustainability of the health care system. According to the interviews in this study, six main themes were established, including 1) objectives, effects, and requirements of the taxation of harmful products, services, and practices, 2) definition, instances, elasticity, and grading of harmful products, services, and practices, 3) Problems in controlling harmful products, services, and practices, 4) controlling harmful products, services, and practices, 5) traffic violations and accidents, and social harms, and 6) tax revenue use and the share of health care.

### Objectives, effects, and requirements of the taxation of harmful products, services, and practices

Taxes on harmful products, services, and practices should be a part of a multi-pronged strategy and action rather than a sole measure for preventing diseases or solving problems. The results of the present study showed that tax measures influence behavioral and social patterns. Also, the effects of taxes on harmful goods should be examined from different aspects, and the objectives of these taxes, especially the promotion of public health, should be considered more than before. Immurana et al. [57] posited that, since the negative health effects of tobacco and alcohol transcend beyond only tobacco/alcohol-related deaths, there is the need to investigate the effect of taxes on harmful products on the health of the entire population.

According to Sassi et al. (2018), Price changes affect the consumption and expenditure of high-income households more than low-income households [18]. Also, a report by the WHO showed that increasing cigarette taxes raises the total cost of smoking for rich people [58].

Tax policies between 1990 and 2017, have had beneficial effects on reducing the consumption of harmful goods in many countries of the world [7]. A 2008 study in the United States of America (USA) demonstrated that higher taxes reduced smoking, especially among the less educated and low-income families [59]. Also, surveys have indicated that taxes on SSBs, reduce purchasing and consumption of these products [60–70].

On the other hand, the present study showed that taxing harmful products, services, and practices, as well as increasing their prices, can encourage or force manufacturers to modify their production lines and improve the quality of their products. Studies have also shown that taxes can encourage industries to improve processes and produce quality products [71, 72].

A common criticism of levying taxes on food products is that it will have a greater impact on low-income households (which have to spend more of their income on food) than on high-income households [73]. The results of this study also show that imposing taxes on those harmful products that are commonly used by people will be problematic for many families, even forcing them to cut some of their main living expenses to afford those products.

To determine the effects and assess the taxation on harmful products, services, and practices, the government should conduct research before implementing tax programs in order to compare the results obtained after tax.

### Definition, instances, elasticity, and grading of harmful products, services, and practices

The examples of unhealthy products must be identified according to the realities of society. In taxation, households’ financial capability and the effects of taxes on consumption in different periods, as well as the elasticity of goods, should be taken into account. According to studies, the rate of demand reduction and who is most affected by prices depend on consumers’ price elasticity of demand [74]. On the other hand, demand for most foods is not elastic, and industries and retailers can shift a large part of the price increases to consumers without significantly reducing consumption [75]. Demand for cigarettes is also inelastic [76]. Because of the inelasticity of demand, experts believe that price increases should be significant to generate meaningful behavioral changes [76, 77]. For example, the WHO has recommended the tax rate on SSBs should be at least 20% [78].

On the other hand, to improve the level of health, policymakers consider labeling goods as a means to change consumer behavior [79, 80]. This study also showed that in addition to pricing tools, guiding consumers to buy healthier products and grading goods based on the type and severity of their effects are good approaches to prevent disease and promote health.

### Problems in controlling harmful products, services, and practices

Such taxation policies will also face many challenges and problems, the most important of which are poor decision-making/planning/execution, production-related problems, smuggling/poor supervision, increased consumption of harmful products, and conflicts of interest. These problems need to be studied in partnership with relevant government agencies, especially the ministries of Health, Industry and Mine, Economy, and Agriculture, to determine the best approach to address and solve them. According to the study by Tamir et al., barriers related to these taxes include opposition of various sectors, technical and bureaucratic obstacles to tax implementation, problems in defining taxes on products, and opposition of the treasury to earmark tax revenue for health education [81].

To control harmful goods and measures, the opposition and resistance of some industries to the taxation of harmful products must also be controlled. For example, price adjustments to promote healthy diets may be opposed by many stakeholders [8]. Ross et al. (2017) showed that the tobacco industry implements seven strategies and actions to oppose and counteract the effects of tobacco tax, including stockpiling, changing product attributes or production processes, lowering prices, over-shifting prices, under-shifting prices, the timing of price increases, and engaging in price discrimination and/or offering promotions [82]. The present study also showed that some industries are opposed to increasing taxes on harmful goods.

### Controlling harmful products, services, and practices

To improve the effectiveness of tax measures in controlling harmful products and services, on the one hand, more work needs to be done on providing high-quality, healthier alternatives, and, on the other hand, serious attention should be paid to all factors that may affect supply and demand (especially prices), people’s purchasing power/beliefs/habits/culture, advertisements, and the quality of products. There is scientific evidence suggesting that the environment in which people choose their diet and their ability to afford healthy foods have huge impacts on their diet [83].

In addition to taxes, the use of complementary measures (such as banning advertising and banning the use of products such as tobacco in public) can help to better deal with these goods [76]. Allocating subsidies for healthy food, improving public health awareness, accessibility to healthy foods, creating a health-promoting environment, and proper nutrition in schools and workplaces are some of the most important recommendations to better deal with harmful goods [84]. The results of the present study also emphasize performing complementary measures, especially banning advertising and raising public awareness.

Also, in our study, it was shown that to deal with the opposition of some industries, the government and the parliament should respond appropriately to face these oppositions and increase the supervision of industries and markets while setting the necessary laws for collecting taxes and considering the executive guarantee of these laws. According to the study by Ross et al., monitoring and analyzing the industry behavior will develop tax performance [82].

### Traffic violations and accidents, and social harms

Annually, traffic accidents cause the death of 1.35 million people in the world and are one of the main causes of injuries, disability, and deaths [85]. Statistics show that 80 to 90% of driving accidents are caused by drivers’ operational mistakes, errors, misbehaviors, carelessness, fatigue, drowsiness, and distraction [86–90]. On the other hand, increasing behavioral and social harms and deviances can cause serious injuries to people in society, especially adolescents and young people. Statistics show that the incidence of these anomalies has increased in recent years [91]. The increase in life and financial costs due to violations, traffic accidents, and social harms were also pointed out in our study, and the need to reduce these costs using financial measurement was emphasized.

The scope of the taxation of harmful measures can be expanded to include traffic incidents and social deviances. Examples and the way taxes and tolls or fines are imposed on traffic violations, behavioral violence, and social anomalies must be properly identified. These fines should be a deterrent and reduce the possibility of violations and damages. Abroodi et al. (2018) showed that immediate response and severe punishment have a positive effect on preventing violations and accidents. Therefore, the legislator’s behavior in dealing with traffic violations and crimes has a significant effect on deterring and controlling them [92].

### Tax revenue use and the share of health care

The revenues generated from the taxation of harmful products and practices must be spent in the right places. The bulk of these revenues should go to agencies and organizations that work in the area of public health.

Since the financial resources will be limited, the allocation of these resources in the healthcare sector should be done with great care and attention to detail, as well as with an emphasis on improving the financial sustainability of the healthcare system. This sustainability will ensure that services remain available to all applicants without uncertainty and interruption [93]. Allocating tax revenues should be considered, especially for health promotion activities, treatment and prevention of diseases, education of people, and subsidizing healthy food [81]. The results of this study also suggest that the tax revenues allocated to the health care sector should be spent carefully and with proper supervision. These revenues should be mainly used in the area of hygiene and preventive health, but they can also be used for culture building, advertising the benefits of healthy products over harmful products in the media, and promoting public sport (A sport that all people can participate in it, Such as walking, cycling, mountain climbing, and public running).

### Recommendations

Regarding research findings, the following recommendations are suggested in the field of tax on harmful products, services, and practices:

- The accurate classification and grading of harmful goods and practices and the reasonable determination of tax rates according to the type of product and its effects on health.

- Paying enough attention to all harmful goods listed by the Ministry of Health, as well as providing suitable alternatives for these goods.

- Accurate and purposeful policy-making and planning to impose taxes, fees, and fines on traffic violations and accidents, and social harms and paying enough attention to the correct implementation of related laws and programs.

- Paying attention to the elasticity of goods and services and considering the impact of taxes on consumption among income groups and different periods.

- Paying serious attention to the prohibition of the advertisement of harmful goods and services, especially through public media.

- Proper and purposeful planning for the correct allocation and use of health-specific tax revenues (such as taxes on harmful goods), prioritization and transparency in the expense of these revenues, as well as adequate and regular monitoring of how resources are used to prevent wasting resources.

- Culture building and raising public awareness about the effects of products, services, and practices that are harmful to health and attention to all determinants of supply and demand.

- Investing in healthy industries and products, modernizing equipment, and considering tax incentives and exemptions for healthy goods or subsidizing these goods.

### Strengths and limitations

The present study had several strengths. The study did not only focus on the use of tax measures in managing harmful products and services but also harmful practices. Moreover, the study used a qualitative approach which helped in providing a deeper understanding of experiences, phenomena, and context, as well as extending knowledge and understanding of realities. The most important limitations of the present study were the concurrence of the study with the COVID-19 pandemic (especially the limitations for face-to-face interviews) and possibly the inadequate survey of some aspects (elasticity of demand for harmful goods and services, traffic violations, behavioral violence, and social anomalies).

## Conclusion

Taxation can serve as a policy and management tool to influence people’s ability and motivation to purchase harmful products and services and as a deterrent against certain harmful practices and activities. Despite the existing limitations and problems, taxation of harmful products, services, and practices within the framework of the specific rules and programs must be seriously pursued. To improve public health, many strategies require proper policy-making, legislation, planning, implementation, and monitoring, especially in the field of financing, prioritization reform approaches, and cooperation and coordination on health issues. Taxes on harmful products, services, and practices could be the most important requirements and facilitators in this regard.

It is suggested that in future studies, important issues such as the implementation, receipt, allocation, and use of taxes on harmful products, services, and practices are examined in more detail. In addition to tobacco, alcohol, and foods containing high salt/sugar and SSBs, other harmful products should also be studied.

## Data Availability

Since the present study is a qualitative study, based on an agreement on the informed consent form, the participants only allowed the researchers to publish the findings as a whole. Therefore, we do not have permission to provide research data and materials in public or in an appendix. However, the research team is committed to providing some data if requested by other researchers by email to the first author (mahdi19205@gmail.com) and corresponding author (hdargahi@sina.tums.ac.ir).

## Abbreviations

INTA: Iran National Tax Administration
MD: Medical Doctorate
MPs: Members of the Parliament
WHO: World Health Organization
SSBs: Sugar-Sweetened Beverages
NCDs: Non-Communicable Diseases
USA: United States of America
